# Tuberculosis services during the COVID-19 pandemic: A qualitative study on the impact of COVID-19 and practices for continued services delivery in Eswatini

**DOI:** 10.1016/j.puhip.2023.100405

**Published:** 2023-07-01

**Authors:** Victor Williams, Alinda G. Vos-Seda, Marianne Calnan, Lindiwe Mdluli-Dlamini, Samson Haumba, Diederick E. Grobbee, Kerstin Klipstein-Grobusch, Kennedy Otwombe

**Affiliations:** a Julius Global Health, Julius Center for Health Sciences and Primary Care, University Medical Center Utrecht, Utrecht University, Utrecht, the Netherlands; b National Tuberculosis Control Program, Manzini, Eswatini; c Division of Epidemiology and Biostatistics, School of Public Health, Faculty of Health Sciences, University of the Witwatersrand, Johannesburg, South Africa; d Ezintsha, Faculty of Health Sciences, University of the Witwatersrand, Johannesburg, South Africa; e University Research Co. LLC, Manila, Philippines; f Center for Global Health Practice and Impact, Georgetown University Medical Center, Washington, DC, USA; g Institute of Tropical Medicine, University of Tübingen, Tübingen, Germany; h Perinatal HIV Research Unit, Faculty of Health Sciences, University of the Witwatersrand, Johannesburg, South Africa

**Keywords:** Tuberculosis, Pandemic, COVID-19, Primary care, Tuberculosis outcomes

## Abstract

**Objectives:**

To describe the impact of the COVID-19 pandemic on tuberculosis services and the different approaches healthcare workers adopted to ensure continued tuberculosis service delivery in Eswatini.

**Study design:**

This is a qualitative study with a cross-sectional design.

**Methods:**

Thirteen nurses and 9 doctors who provide tuberculosis care from 10 health facilities participated in an in-depth interview to describe how the COVID-19 pandemic affected tuberculosis services and the approaches adopted to ensure continued patient care. Twenty in-person and 2 telephone interviews were conducted. The participating facilities were selected based on a ranking criterion of the number of patients seen. Data were analyzed using thematic content analysis. NVivo 12 software was used for qualitative analysis, and the Consolidated Criteria guided the study for Reporting Qualitative research (COREQ).

**Results:**

Two major themes emerged: COVID-19 impacted services delivery and access; and best practices that ensured healthcare services delivery. Six sub-themes describe how COVID-19 impacted services: all attention focused on COVID-19; COVID worsened the health system challenges; COVID hindered patients from accessing care; patients defaulted due to the lockdown; COVID impacted the quality of care and increased the risk of infection among healthcare workers. Five sub-themes describe best practices that ensure continued service delivery: Home-based care, Patient support, Patient Education, Integrated Services, and Staff rotation.

**Conclusion:**

While various strategies were adopted globally to mitigate the impact of the COVID-19 pandemic, these strategies need contextualization to be effective and sustainably incorporated into routine care to ensure continuity of and access to TB and other healthcare services.

## 1 Introduction

At the peak of the COVID-19 pandemic in 2020, tuberculosis (TB) case notifications dropped by as much as 47% [1]. Many TB high-burden countries continue struggling to recover as newer COVID-19 variants arise and impact resource allocation and service delivery [2,3]. Impacts include the closure of health facilities and laboratories, healthcare workers becoming sick, stock out of medical supplies, and repurposing of existing facilities and staff. Additionally, an impact modelling study demonstrated there could be an increase of up to 20% in TB-related deaths due to delayed diagnosis over the next five years [4].

Data from different World Health Organisation (WHO) regions indicate access to other health services including HIV, malaria, vaccination, non-communicable diseases, and mental health were affected [[5], [6], [7], [8]]. The responses to address the impact of COVID-19 on these services including TB varied by country, especially noting wide differences in health systems and existing infrastructure before the pandemic. Associated with the public health response was the economic meltdown caused by the global shutdown and restriction in both local and international movements to limit new infections [9].

Like other countries, the declaration of COVID-19 as a global pandemic in March 2020 by WHO activated a national response in Eswatini which mandated the use of face masks, hand washing and sanitization, closure or limited operations at some institutions and the general restriction in movement [10]. These measures affected access to healthcare including TB services. In this paper, we describe the impact of the COVID-19 pandemic on TB service delivery and different practices adopted by healthcare workers in Eswatini to ensure continued services.

## 2 Methods

### 2.1 Context

TB services in Eswatini are coordinated by the Ministry of Health (MOH) through the National Tuberculosis Control Programme (NTCP) [11]. The NTCP oversees TB services through a network of primary care clinics, health centres and regional hospitals where every patient is screened for TB regardless of the primary complaint. Drug-sensitive TB patients are managed at the primary care clinics while those with multi-drug resistant TB are initially treated at one of the regional hospitals and down referred to the primary care clinic when they achieve sputum conversion.

### 2.2 Study design and setting

This qualitative study, evaluated cross-sectionally, is based on the socio-ecological framework and is part of a prospective cohort study which has been described earlier [12]. Healthcare workers from eleven health facilities in Eswatini were interviewed to identify barriers to TB and integrated TB–DM services, the effect of COVID-19 on TB and TB-DM services and the practices adopted to ensure sustained services delivery. This article focuses on the effect of COVID-19 on TB services and the different practices adopted by healthcare workers to ensure sustained TB services. The research team consists of specialist physicians and epidemiologists (AV, MC, SH, DEG, VW), a nurse practitioner in the national TB program (LM), a biostatistician experienced in quantitative and qualitative TB research (KO), an epidemiologist and global health researcher (KKG).

### 2.3 Data collection

The eleven health facilities selected for this study consist of five hospitals, one health centre and five primary care clinics across the four regions in Eswatini. The facilities were purposively selected based on a ranking criterion of the highest number of patients two quarters before the study. Doctors and nurses were invited to participate if they had worked at the health facility for a minimum of one year and provided care for TB patients. Those who met the inclusion criteria and provided informed consent were included.

Interviews were conducted between May and June 2022 by a trained research assistant assisted by the principal investigator at the clinic. Participants informed consent included consent to record the interviews. One participant declined to consent to the recording and interview notes were taken. Participants received an orientation on the study and a study information sheet. In-person interviews were conducted with 20 participants and two participants were interviewed telephonically. A semi-structured interview guide (Supplementary File 1) was used for the interview. Each interview lasted 30–45 minutes and healthcare workers used the language of their preference (English or Siswati). The study was approved by the Eswatini Health and Human Research Review Board (EHHRRB-036/2021).

### 2.4 Data analysis

Each interview was transcribed immediately afterwards without linking it to the healthcare worker. The research assistant and the study principal investigator reviewed each transcript against the interview recording to ensure the accuracy of the transcript. Inductive and deductive codes were identified from the transcripts and documented in a codebook (VW). Similar codes in the codebook were grouped into sub-themes and similar sub-themes were grouped into major themes for the study (VW, MC). Three research team members reviewed the code book and the themes for accuracy and consistency. Inaccuracies in the codes, sub-themes, and themes were resolved in a study meeting. Reviewing the codes was iterative to ensure the finalized themes were accurate. The results were presented using text analysis. NVivo 12 software was used for qualitative analysis and the Consolidated Criteria for Reporting Qualitative Research (COREQ) guided the reporting of this study [13,14] (Supplementary file 2).

## 3 Results

### 3.1 Characteristics of respondents

Twenty-two healthcare workers from ten healthcare facilities participated in the interview (Table 1). Although two participants from one health facility were not available for interviews, data saturation was achieved.

**Table 1:** Characteristics of study participants.

Variable	Overall (n = 22)
**Age (years)**	
Median (Q1, Q3)	36.0 (33.0, 43.0)
Min, Max	26.0, 59.0
**Gender of healthcare worker**	
Female	9 (41.0%)
Male	13 (59.0%)
**Highest qualification attained**	
Certificate	1 (5.0%)
Degree	10 (41%)
Diploma	4 (18.0%)
Postgraduate	8 (36.0%)
**Occupation**	
Medical Doctors	9 (41.0%)
Nurses	13 (59.0%)
**Number of years providing care for TB patients**	
Median (Q1, Q3)	4.0 (3.0, 7.0)
Min, Max	1.0, 15.0

Data from the interviews indicate two themes which describe the healthcare worker's perspectives on the impact of COVID-19 on TB services delivery and access and best practices which enabled services delivery during the COVID-19 pandemic. The different themes and subthemes are summarized in Table 2.

**Table 2:** Themes and subthemes in the study.

Themes	Subthemes
COVID-19 impacted services delivery and access	All attention focused on COVID
	COVID worsened the health system challenges
	COVID hindered patients from accessing care
	Patients defaulted due to the lockdown
	COVID impacted the quality of care
	Increased risk of infections among healthcare workers
Some practices ensured healthcare services delivery	Home-based care
	Patient support
	Patient Education
	Integrated services
	Staff rotation as a best practice

### 3.2 COVID-19 impacted services delivery and access

#### 3.2.1 All attention focused on COVID

Most participants stated many aspects of healthcare delivery were deprioritized at the start of the COVID-19 pandemic. Services were implemented partially or suspended.

“*More attention was given to COVID-19, sputum was not collected due to COVID-19 hence many patients who were TB+ were lost in the process since patients were only screened mostly for COVID-19. Many patients who came with NCDs during the COVID-19 phase missed out because we were afraid of conducting some of the tests*” R1

“*Sputum induction was stopped due to the pandemic since we were in fear of the exposure to COVID-19*” R2

#### 3.2.2 COVID worsened the health system challenges

Respondents observed that existing health system-related challenges worsened during the pandemic including disruptions in the supply of drugs and consumables and shortages of healthcare workers (Table 3).

**Table 3:** Illustrative quotes from the participants in the study.

Sub-theme	Quotes
3.2.2 COVID worsened the health system challenges	“… much attention was given to COVID-19 such that other medications were out of stock which hampered the control of the chronic conditions, at times patients were getting their medications at the comfort of their homes without a medical practitioner to ensure if their condition is getting better or worsening” R5
	“The availability of drugs during COVID-19 was also a challenge from the suppliers as well.” R11
	“… most patients who screened positive for COVID-19 were also sent to the TB department, hence they were flooding the department... Both the confirmed and the presumptive TB cases are seen by the same person due to the associated symptoms of TB and COVID-19… we were seeing that challenge of the shortage of staff during COVID-19 surge …” R21
3.2.4 Patients defaulted due to the lockdown	“… what happened is that patients were not coming to the facility hence we were missing cases, and some patients were defaulting.” R9
	“During COVID-19 many of our patients defaulted and we had many lost to follow-up due to lockdown and the travel restrictions which were put in place. There was no transport to do home-based care visits” R17
	“When COVID-19 started, movement was restricted hence it affected the patients who were coming to our facility*”* R20
3.2.5 COVID impacted the quality of care	“Due to COVID-19 we were limiting the amount of time the patient would spend in the facility, such that we were not checking the blood pressure, even some processes that were followed before were not followed in the COVID era such that it was easy to miss a TB patient who has COVID or even misdiagnose a patient.” R3
	“The standard of care to patients declined especially if a patient was diagnosed with COVID-19, thus the time spent with the patient was reduced” R7
	“Since COVID and TB almost share the same symptoms, all the TB and COVID patients are reviewed by one nurse which has increased the volume (of patients) seen by the nurse thus increasing the waiting period as well.” R20
	“We normally face delayed transportation of samples to the national lab... The ambulance is not always available for emergencies which can affect the standard of care” R19
	“… we sometimes have a problem with our GeneXpert machine and sometimes we run out of TB-lam… There is no proper follow-up on culture, sometimes we send, and the results do not come back” R20
3.3.2 Patient support	“… we have our treatment supporter who also provides counselling to our patients… we do provide transport money to our patients so that they cannot be frustrated when it’s time for refills …” R8
	“COVID-19 did affect us but not that much, most of our TB patients were coming and if they didn’t come, we call them, and if they are far, we used their nearest facility for refills” R12
	“… We are also giving food parcels to our patients who are both TB and HIV positive to improve their nutrition because most of our patients are struggling with nutrition” R21

#### 3.2.3 COVID hindered patients from accessing care

With the uncertainty around COVID-19, most chronic care patients stopped visiting health facilities to avoid infection, hindering routine care, and increasing their risks of poor treatment outcomes.

“… *but they (patients) were afraid to come to the clinic because they thought they would contract COVID-19 from the facility*” R5

“*Patients were not coming to the clinic for monitoring and check-ups due to fear of contracting COVID, this led to other patients being severely attacked by TB and others had their DM escalated*” R7

#### 3.2.4 Patients defaulted due to the lockdown

The respondents observed that some patients, besides not visiting the health facilities, stopped taking their medications while others could not be reached or contacted (Table 3). These losses to follow-up were, attributed to the different COVID-19 prevention measures and limited resources at some health facilities to provide alternative means of care such as home visits.

#### 3.2.5 COVID impacted the quality of care

Some of the COVID-19 infection prevention measures instituted at different health facilities may have impacted the quality of care provided by healthcare workers (Table 3). Limiting the services available, restricting the number of patients that are reviewed daily and increased time before accessing a service due to patient screening and triaging contributed to decreased quality of care.

#### 3.2.6 Increased risk of infections in healthcare workers

Respondents had reservations about interacting with patients as they were at an increased risk of infection with COVID-19. Some of their colleagues nonetheless got infected, reducing the number of staff available for patient care, and further impacting the quality of care negatively.

“*Sputum induction was stopped due to the pandemic since we were in fear of the exposure to COVID-19*” R2

“*We were afraid of the patients because we were afraid of contracting COVID-19 from them*” R10

“*One can also talk about the issue of human resources during COVID-19 since many of our staff were affected (infected) this affected the standard of care for patients*” R19

### 3.3 Some practices ensured healthcare services delivery

#### 3.3.1 Home-based care

An adaptation to ensure continuity of services for patients was community and home-based care. Patients received essential medical care and medication refill for chronic conditions within their community, at home, or at a pre-arranged service delivery point.

“*We usually do community and home visits wherein we do TB contact tracing and TB history; this helps us to uncover TB presumptive cases*” R6

“… *we are doing home visits to deliver their medication to relieve them with the transport fare* …” R21

#### 3.3.2 Patient support

The respondents also provided different types of support for patients during the pandemic to encourage them and to ensure they continue with their treatment (Table 3). Some of these include providing counselling, transport reimbursement to the clinic, follow-up calls, drug refills near their homes and food supplies.

#### 3.3.3 Patient education

The respondents felt health education was important to empower their patients to become responsible for their health and minimize TB infections within the community.

“*We have dedicated our Wednesdays… for teaching and review of the patients. TB treatment also involves teaching the patient about health education and how to prevent the spread of TB even at home*” R10

#### 3.3.4 Integrated services

Offering services at different sections of a health facility can prolong the time a patient spends at a health facility and requires more personnel. This may discourage some patients from accessing care. To avoid this, the respondents indicated they introduced a system where possible services a TB patient will require during a visit are provided at one service point. This will reduce waiting time, encourage patients to keep their appointments and minimize required staff.

“*We have introduced the one-stop-shop, wherein we have integrated all the services at one point*” R15

#### 3.3.5 Staff rotation as a best practice

Limited staffing and skillset are challenges at most health facilities. This was obvious at the peak of the pandemic when several healthcare workers were infected and had to isolate. The respondents stated they had a process where staff were intermittently moved to provide services at other units to avoid delay or suspension of services.

“*To ensure continual service provision we do staff rotation, if a TB nurse is not available, one is always assigned to the TB department*” R4

“*Yes, we have enough clinical staff. We normally rotate so that whenever there is a gap someone can cover it up*” R9

## 4 Discussion

Based on the views of healthcare workers, we describe some of the impacts of the COVID-19 pandemic on TB service delivery and access in Eswatini; and some approaches by healthcare workers to ensure the continuity of TB services. At the height of the pandemic, COVID-19 took priority over other conditions, worsening health system challenges which were already in a precarious state. Different measures adopted to limit the spread of COVID-19 infection hindered patients from accessing care as some services were suspended or limited. This affected the number of patients accessing care and the quality of services provided by healthcare workers. Healthcare workers were equally affected by the pandemic, resulting in reduced number of staff for patient care. Despite these challenges, healthcare workers adopted different methods to ensure patients continued receiving care. These included providing care for patients within their communities or at home; providing additional support to patients such as counselling, transport, food packages, and health education; making different services available at one service point and adopting staff rotation to ensure a healthcare worker is always available to attend to patients.

### 4.1 Comparison with other studies

Available evidence at the onset of the pandemic indicated that COVID-19 would adversely affect TB services delivery resulting in reduced access by different population groups with poor outcomes [4,15,16]. This was corroborated by the WHO Global Tuberculosis Report 2021 on COVID and TB which showed that different TB targets were missed between 2019 and 2020 when the pandemic commenced compared to the years 2017–2019. For instance, there was an 18% reduction in global TB case notification with a lower mean TB case notification in most TB high-burden countries notably in Gabon (80%), the Philippines (37%) and Lesotho (35%) [7]. There were also reductions in people commencing MDR/RRTB treatment, people being initiated on TB preventive therapy, a reduction in coverage of the bacille Calmette-Guérin (BCG) vaccine in children and a reduction in spending for TB prevention, diagnostics and treatment services [7,17]. A recent study from Eswatini shows TB case notifications decreased during the pandemic compared to the period prior. Death rate increased to 21.3% compared to 10.8% and the odds of unfavourable outcomes were higher (aOR 2.91, 95% CI: 2.17–3.89) during the pandemic compared to the period prior [18].

Findings from this study indicate a complex interaction of patient-level, socioeconomic, and health system factors with limited emergency response capability. These, coupled with the urgent need to control a pandemic caused by a pathogen whose epidemiology was not fully understood vastly accounted for the reduced access to TB services; further contributing to the global reduction in TB achievement. A Nigerian survey describing TB and COVID-19 screening by healthcare workers during the lockdown indicates that 54% of healthcare workers were not screening patients for TB during this period [19]. Similarly, a review of TB services in India during the COVID lockdown indicates there was a widespread disruption in services at both the primary and secondary health facilities; and that different health guidelines aimed at limiting COVID virus transmission limited access to TB services [20]. This is similar to what we have reported as some services were temporarily suspended while others operated at half capacity. Reports from Sierra Leone [21], the United States [22] and Portugal [23] confirm similar findings. In a multi-country cross-sectional survey, about 40% of respondents indicated it was more difficult for HIV and TB patients to access a health facility during the COVID pandemic and another 31% indicated access to TB patient support such as food and counselling was interrupted [24].

Another critical factor was the repurposing of TB resources for COVID-19 including the equipment GeneXpert used for TB diagnosis [17,25]. This negatively impacted TB diagnosis, access to treatment and follow-up care as the number of TB samples that can be processed was reduced. Available human resources and clinic spaces were also reassigned in some instances to provide COVID-19 services. When combined with other related factors such as stock-outs of medications, reduced facility operational hours and movement restrictions, fewer patients access services [17]. Several healthcare workers who were also the first responders got infected with COVID-19 [26]. While some died, others suffered different forms of physical, mental and psychological impacts including fear, anxiety and depression [[27], [28], [29]]. This was similarly reported in our study as healthcare workers were afraid of seeing patients due to fear of infection. This also contributed to a reduction in the quality and number of services provided. While the reduced number of healthy staff could have been responsible for the reduced quality of care, limited availability of personal protective equipment (PPE) and limited support to healthcare workers contributed to sub-optimal service delivery [17,30].

Due to the pandemic, more people globally have become poorer [9]. TB already being a disease associated with poverty means more TB patients are disadvantaged. In addition to the increased poverty level, measures aimed at controlling the pandemic hindered patients from accessing care and basic support including food and psychosocial services; and transport to health facilities by both patients and healthcare workers [17,24]. Patients in Eswatini also encountered these different challenges and healthcare workers adopted different measures to continue providing services. While these measures – home-based care, patient support services and patient education may not be completely new, their adaptation within limited resources ensured patients remained in care. This is different compared with some high-income countries where patients could easily access healthcare through telemedicine consultations, virtual consultation and monitoring [30].

### 4.2 Strength and limitations

Our qualitative study presents healthcare workers' perspectives on how COVID-19 impacted TB service delivery at the peak of the pandemic and the different steps they took to ensure continued service delivery in Eswatini. This will provide insight into the reduced performance in key TB outcomes in Eswatini and other LMICs and guide TB program implementers on measures that can increase access to TB services. Our study participants were drawn from health facilities across Eswatini so our findings are representative of how COVID-19 impacted TB services. As a limitation, our study does not present data to quantify the impact of the pandemic on key TB indicators. This would have provided a more objective view of the problem. Secondly, we interviewed only doctors and nurses. We did not include other healthcare workers such as community TB officers responsible for active TB case finding, TB treatment supporters, and laboratory and pharmacy technicians. These healthcare workers could have presented a more complete perspective of how the pandemic affected TB service delivery and patients. Finally, we did not interview TB program staff. They would have offered an additional explanation for observations by our participants.

## 5 Conclusion

The COVID-19 pandemic affected health systems globally, even in advanced economies. It is vital different approaches that sustained services are standardized so patients can continue receiving care. In the future, tailored and hybrid approaches to care should be developed where patients can access care either at the health facility or remotely with the freedom to visit a health facility if necessary. Due to limited infrastructure, approaches such as telemedicine for consultation may not be feasible immediately in most LMICs but telephone calls, SMS and home-based services can be used for medication dispensing, testing, follow up and psychosocial support. Finally, bidirectional screening and integration of care for different comorbidities can increase access to care.

## Ethical approval

Eswatini Health and Human Research Review Board (EHHRRB) (Ref: EHHRRB 036/2021).

## Funding

VW is funded by the 10.13039/100006090Global Health PhD Support Programme at the University Medical Center, Utrecht, The Netherlands.

## Author contributions

VW conceptualized the study and wrote the protocol under the supervision of AV, DEG, KO, and KKG. DEG and KKG sourced funding for the research. VW, SH, MC, and LD obtained approvals and conducted the study. VW, AV, and KO conducted the analysis. VW and MC wrote the first draft. All authors reviewed the results, contributed to the interpretation, and reviewed the different drafts of the manuscript. All authors reviewed and approved the final draft of the manuscript.

## Data availability

The data used for this study is available and will be shared by the corresponding author upon request.

## Declaration of competing interest

None declared by the authors.
